# Pins Gene Table v2.0: An Online Genome Database of 37 *Pythium insidiosum* Strains for Gene Content Exploration and Phylogenomic Analysis

**DOI:** 10.3390/jof10020112

**Published:** 2024-01-29

**Authors:** Weerayuth Kittichotirat, Preecha Patumcharoenpol, Thidarat Rujirawat, Sithichoke Tangphatsornruang, Chompoonek Yurayart, Theerapong Krajaejun

**Affiliations:** 1 Bioinformatics and Systems Biology Program, School of Bioresources and Technology and School of Information Technology, King Mongkut’s University of Technology Thonburi, Bangkhunthian, Bangkok 10150, Thailand; weerayuth.kit@kmutt.ac.th; 2 Systems Biology and Bioinformatics Research Group, Pilot Plant Development and Training Institute, King Mongkut’s University of Technology Thonburi, Bangkhunthin, Bangkok 10150, Thailand; 3 Interdisciplinary Graduate Program in Bioscience, Faculty of Science, Kasetsart University, Bangkok 10900, Thailand; preecha.pa@ku.th; 4 Research Center, Faculty of Medicine, Ramathibodi Hospital, Mahidol University, Bangkok 73170, Thailand; thidarat.ruj@mahidol.ac.th; 5 National Center for Genetic Engineering and Biotechnology, National Science and Technology Development Agency, Pathum Thani 12120, Thailand; sithichoke.tan@nstda.or.th; 6 Department of Microbiology and Immunology, Faculty of Veterinary Medicine, Kasetsart University, Bangkok 10900, Thailand; fvetcny@ku.ac.th; 7 Department of Pathology, Faculty of Medicine, Ramathibodi Hospital, Mahidol University, Bangkok 73170, Thailand

**Keywords:** *Pythium insidiosum*, pythiosis, gene table, genome, phylogenomics

## Abstract

Unlike most pathogenic oomycetes, *Pythium insidiosum* infects humans and animals instead of plants. *P. insidiosum* has three clinically relevant genotypes/clades that cause a severe disease called pythiosis. To develop strategies for infection control, it is necessary to understand the biology and pathogenesis of this pathogen. Investigating the evolutionary mechanisms behind the host-specific adaptation is vital, and comparative genomic analysis can help with this. To facilitate genomic analysis, an online bioinformatics tool called *P. insidiosum* (Pins) Gene Table v2.0 was developed. This tool includes genomic data from 37 genetically diverse *P. insidiosum* strains and four related species. The database contains 732,686 genes, grouped into 80,061 unique clusters and further divided into core and variable categories at genus, species, and genotype levels. A high-resolution phylogenomic relationship among *P. insidiosum* strains and other oomycetes was projected through hierarchical clustering and core gene analyses. 3156 *P. insidiosum*-specific genes were shared among all genotypes and may be responsible for causing disease in humans and animals. After comparing these species-specific genes to the MvirDB database, 112 had significant matches with 66 known virulence proteins, some of which might be involved in vascular occlusion, which is a pathological feature of pythiosis. The correlation of genotypes, geographic origins, and affected hosts of *P. insidiosum* suggests that clade-I strains are more specific to animals, while clade-II/III strains are more specific to humans. The clade-specific genes might link to host preference. In summary, Pins Gene Table v2.0 is a comprehensive genome database accessible to users with minimal bioinformatics experience for the analysis of *P. insidiosum* genomes.

## 1. Introduction

*Pythium insidiosum* is an oomycete microorganism that belongs to the Stramenopiles/Sar clades of the Eukaryota Superkingdom (https://www.ncbi.nlm.nih.gov/taxonomy (accessed on 10 November 2023)). This organism is responsible for causing pythiosis, a severe infection that can affect humans and animals worldwide [1,2,3,4]. Unfortunately, diagnosing pythiosis can be challenging due to the lack of awareness about the disease and the absence of a reliable and easily accessible test [5,6,7,8,9,10,11,12,13,14]. Regular antifungal therapy is usually ineffective against this infection, so surgical removal of the affected organ (typically the eye or leg) is often necessary to prevent the disease from progressing and leading to death [5,15,16]. Developing a practical approach for detecting and treating pythiosis is crucial. To achieve this goal, we need to gain a better understanding of the biology and pathogenesis of *P. insidiosum*. Unfortunately, this organism remains poorly understood, despite the fact that it is unique among oomycetes in that it primarily infects humans and animals rather than plants [17]. Comparative genomic analysis can reveal the evolutionary mechanisms behind the host-specific adaptation in *P. insidiosum*. Investigating these mechanisms is essential to a better understanding of the pathogen.

In the previous study [18], we introduced the *P. insidiosum* (Pins) Gene Table v1.0 as an online bioinformatics tool to help researchers in the comparative genomic study of this species. The software included the genome contents of 10 strains of *P. insidiosum*, categorized into three phylogenetic clades: three strains in clade-I (prevalent in the Americas), five strains in clade-II (prevalent in Asia and Australia), and two strains in clade-III (mostly prevalent in Thailand) [19]. While this tool was helpful, it only represented a small fraction of the genomic diversity of *P. insidiosum*. To address this limitation, we utilized the high-throughput genome sequencing platform MGISEQ-2000RS (MGI Tech Co., Ltd., Shenzhen, China) to generate additional genome data from 37 *P. insidiosum* strains. These strains were isolated from various hosts and geographic locations, and their genomic data was incorporated into the Pins Gene Table v2.0. We also included the genome sequences of four other oomycetes species (i.e., *Pythium rhizo-oryzae*, *Pythium catenulatum*, *Pythium aphanidermatum*, and *Paralagenidium karlingii*) for comparison with *P. insidiosum*. It is worth noting that *P. rhizo-oryzae* and *P. catenulatum* inhabit the same environment as *P. insidiosum* but do not cause infections in humans or animals [20,21,22]. Identifying species-specific genes could help us understand the pathogenicity of *P. insidiosum*.

Among the genome-related information on *P. insidiosum* reported so far [18,23,24], the Pins Gene Table v2.0 is currently the most comprehensive genome database available. It was developed to promote genomic comparison, phylogenomic analyses, and pathogenicity exploration of this pathogenic species. The software’s tabular format makes it easy to use and understand, even for those with limited experience in bioinformatics programming. In this article, we will describe how the Pins Gene Table v2.0 was developed and provide some examples for navigating, comparing, and analyzing the genomes to better understand the biology and pathogenesis of *P. insidiosum*.

## 2. Methods and Materials

### 2.1. Generation and Recruitment of Genome Data from P. insidiosum and Related Species

Thirty-seven strains of *P. insidiosum* and four related oomycete species (i.e., *P. catenulatum* strain RM906, *P. rhizo-oryzae* strain RCB01, *Pythium aphanidermatum* strain ATCC32230 and *Paralagenidium karlingii* strain CBS134681) maintained in our laboratory collection (Table 1) were analyzed for species identity and clade classification (i.e., clade I, II, and III) using rDNA sequence analysis [19,25]. The organisms were grown in Sabouraud dextrose broth, shaking at 37 °C for 7–10 days. Hyphal materials were separated from the broth culture, and genomic DNA (gDNA) was extracted using the method of Lohnoo et al. [26]. A gDNA library for each organism was constructed using an MGI Eazy FS Library Prep Kit (MGI Tech Co., Ltd., Shenzhen, China) and the manufacturer’s protocol. Finally, shotgun genome sequences were generated through 150-bp paired-end sequencing by an MGISEQ-2000RS sequencer (MGI Tech Co., Ltd., Shenzhen, China). Obtained reads were cleaned using MegaBOLT V2.4.0 before de novo sequence assembly using SPAdes v3.14.0 [27] and the default setting (the k-mer size was adjusted to 21, 33, 55, 77, and 99).

All recruited draft genomes were analyzed for basic parameters, such as total bases, number of contigs, N50, and GC content. Augustus v3.3.3 [28] was used to predict open reading frames (ORFs) in the genomes. Draft genomes from two out of 37 *P. insidiosum* strains (i.e., ATCC90586 and P43SY (also known as Pi057C3)) have been reported elsewhere [29]. All newly assembled genome sequences have been assigned an accession number and deposited in the National Center for Biotechnology Information (NCBI) and DNA Data Bank of Japan (DDBJ) databases.

### 2.2. Clusters of Homologous gene for Genome Content Comparison

We compared the gene content of 37 different *P. insidiosum* strains and four other oomycete species listed in Table 1 to examine their genomic variability. Our previously published protocol [30] was used to group the genes together based on various sequence similarity parameters at both DNA and protein levels. To group genes into the same cluster, we employed the following criteria: BLAST *E*-value of 10^−6^, pairwise sequence identity of at least 30%, and pairwise sequence alignment coverage for both query and subject of at least 50%. These loose criteria enable the grouping of distant homologs and reduce the chances of false positives in detecting group- or genome-specific genes present only in a subset of genomes. If a gene is still not found in a genome using these criteria, it is probably absent rather than present but significantly diverged from its corresponding orthologous genes found in other genomes. The final result of the homologous gene cluster analysis is displayed in a table format, referred to as the Pins Gene Table v2.0. Each row in the table represents a gene, while the columns represent the genomes from all 41 organisms used in this study. Each cell in the table provides information on homologous genes or genomic regions found in the corresponding genome.

### 2.3. Phylogenomic Analysis of P. insidiosum and Other Oomycetes Based on Core Genes

For the core gene-based phylogenomic analysis, we selected 115 genes identified in all 37 *P. insidiosum* strains and four other oomycete species. We ensured that these genes did not have length variations of more than 45% relative to the longest representative gene in each homologous gene cluster. We aligned the nucleotide sequences of each core gene from all genomes using ClustalW version 2 with default parameters [31]. After aligning, we removed gaps and concatenated the results to produce a single multiple-sequence alignment file. We used FastTree2 to create a maximum likelihood tree and carried out a bootstrap analysis to test the reliability of the tree [32]. Finally, the phylogenetic tree was visualized using FigTree v1.4.0 (http://tree.bio.ed.ac.uk/software/figtree/ (accessed on 15 November 2023)).

### 2.4. Hierarchical Clustering of the Gene presence Profile Data

We created gene presence profile data for each of the 41 genomes from our Gene Table result. Specifically for each genome, the value of 1 or 0 was used to represent whether or not each of the gene cluster was found. If a gene cluster was not predicted, but a homologous genomic region was found, we still assigned a value of 1 (present). We then used MeV 4.8.1 with default parameters to perform hierarchical clustering of the genomes based on their gene presence profile data [33]. Finally, we used FigTree software to draw the hierarchical clustering tree result.

### 2.5. Functional Annotation of the Identified Genes

We used our Gene Table result to obtain genes found in all genomes (Core genes) and exclusively identified in *P. insidiosum* but not in other oomycetes (*P. insidiosum*-specific). The protein sequences of these genes were subjected to a BLAST search against the NCBI non-redundant protein database (https://www.ncbi.nlm.nih.gov/ (accessed on 15 November 2023)) and the SwissProt database (https://ftp.uniprot.org/pub/databases/uniprot/current_release/knowledgebase/complete/uniprot_sprot.fasta.gz (accessed on 15 November 2023)). The annotated proteins were assigned into one of the four Clusters of Orthologous Groups (COG) functional categories: (i) information storage and processing, (ii) cellular processes and signaling, (iii) metabolism, and (iv) poorly characterized or hypothetical proteins [34]. The so-called *P. insidiosum*-specific genes were further compared against the MvirDB database [35] to identify any putative virulence factors present in *P. insidiosum* similar to those found in other known pathogens (BLAST *E*-value cutoff of 10^−6^).

## 3. Results and Discussion

### 3.1. Genome Data of P. insidiosum Strains and Related Species

Table 1 summarizes the genome data of 37 *P. insidiosum* strains and four related oomycete species in this study. *P. insidiosum* was collected from humans (*n* = 18), animals (i.e., horses and dogs; *n* = 14), and the environment (*n* = 5); they represent the clinically relevant genotypes of the pathogen, including clade-I (primarily prevalent in the Americas; *n* = 11), clade-II (mostly prevalent in Asia and Australia; *n* = 14), and clade-III (prevalent in Thailand; *n* = 12). The clade-I strains of *P. insidiosum* are strongly associated with pythiosis in animals, while the clade-II and -III strains can infect humans and animals. Among the closely related oomycetes, *P. karlingii* and *P. aphanidermatum* can occasionally infect humans or animals [36,37,38]. *P. rhizo-oryzae* and *P. catenulatum* [20,21,22] were included in this study for genome comparison because they inhabit the same environment as *P. insidiosum*. However, there is no report of their infection in humans or animals.

This study employed the MGI short-read platform for genome sequencing of all the organisms. The gDNA samples extracted from all organisms were quantity- and quality-validated to ensure the genome data obtained were comparable. The genome sizes and the number of predicted genes in *P. insidiosum* appeared diverse, ranging from 42.7 to 75.4 Mb (average: 59.8 Mb) and 12,008 to 22,015 genes (average: 17,006 genes), respectively. The contig sequences were compared with the NCBI nucleotide database, and no significant hit with other species was detected. This suggests that the generated genome sequence data did not possess any contamination. *P. insidiosum* clade-III strains had a larger average genome size (63.9 Mb), followed by clade-I (60.5 Mb) and clade-II (55.6 Mb) strains. The clade-II strains possessed a markedly lower gene number (16,202 genes on average) compared with clade-I (17,384 genes) and clade-III (17,598 genes) strains. The non-pathogenic *Pythium* species (i.e., *P. rhizo-oryzae* and *P. catenulatum*) had a remarkably larger genome size (97.4 Mb on average) and higher gene number (41,901 genes on average) compared with *P. insidiosum* (59.8 Mb and 17,006 genes on average). In contrast, two other oomycete species (i.e., *P. karlingii* and *P. aphanidermatum*), which occasionally infect humans or animals, had a compatible genome size (average: 58.3 Mb) but a strikingly lower gene number (average: 9825 genes) compared with *P. insidiosum*.

Despite the variation in genome sizes and gene numbers, the GC contents within *P. insidiosum* were similar, ranging from 56.9 to 57.7%, with an average of 57.4%. The other recruited oomycete species showed relatively lower GC contents (<54%) in *P. karlingii* and *P. aphanidermatum* and relatively higher GC contents (>59%) in *P. rhizo-oryzae* and *P. catenulatum*. The GC content of 57–58% could be considered a characteristic of *P. insidiosum*. The differences in genome sizes and gene contents among *P. insidiosum* strains and related oomycete species might link to the different phenotypes and virulence.

### 3.2. Classification of Homologous Genes within the Oomycetes

A total of 732,686 genes can be identified in the genomes of *P. insidiosum* (37 strains) and other recruited species (i.e., *P. catenulatum*, *P. rhizo-oryzae*, *P. aphanidermatum*, and *P. karlingii*), as shown in Table 1. These genes can be grouped into 80,061 unique homologous gene clusters using a stringent threshold, such as BLAST *E*-value of 10^−6^, sequence identity of at least 30%, and sequence alignment coverage of at least 50%. All homologous gene cluster data were stored in the Pins Gene Table v2.0, which can be accessed online at https://202.28.6.19/cgi-bin/gt/viewer?organism=pythium&build=211019 (accessed on 15 January 2024) and can be operated using the explicit instruction of the previous software version [18,39]. The number of genes in any single genome (ranging from 12,008 to 22,015) was markedly smaller than the number of homologous gene clusters (*n* = 80,061), indicating that no single genome had its genes classified in all clusters. For instance, the genome of *P. catenulatum* RM906 contained the highest gene number (*n* = 47,975), which can be allocated into 28,199 clusters (35.2% of all clusters). The genome of *P. insidiosum* P52WN contained 22,015 genes (the highest among this species), which can be assigned to only 10,417 clusters (13.0% of all clusters). These findings suggest a high variation in the gene contents among these microorganisms, especially within *P. insidiosum* strains.

### 3.3. Gene Contents of P. insidiosum and Other Oomycetes

**Figure 1:**
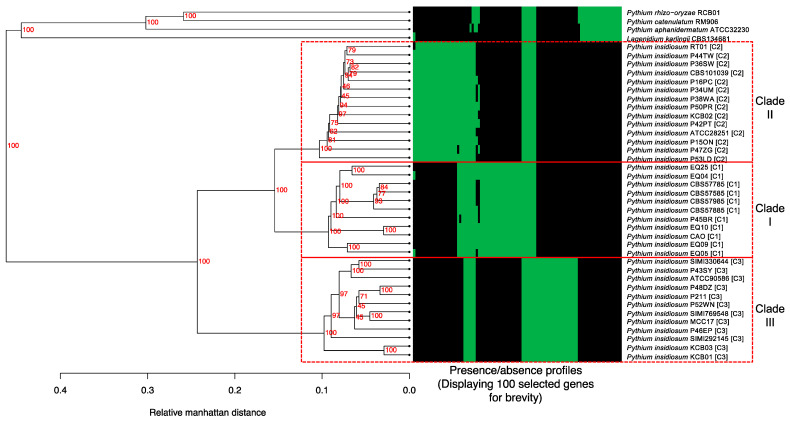
Hierarchical clustering analysis of gene presence/absence profiles in 37 *P. insidiosum* and four related oomycetes, based on 80,061 homologous gene clusters identified across all organisms. The derived dendrogram illustrates the genetic relationships among the organisms, with bootstrap values indicating the confidence of each node (displayed as red numbers). The heat map shows the presence (green) or absence (black) of 100 selected genes correlating with the dendrogram. The red boxes highlight the rDNA-based genotypes (i.e., Clade-I, -II, and -III) of *P. insidiosum*.

To examine the similarities and differences in gene content among *P. insidiosum* strains and other oomycete species, we analyzed the presence and absence of 80,061 homologous gene clusters across their genomes. The gene presence/absence profiles were used to perform a hierarchical clustering analysis to obtain a dendrogram, which revealed a clear separation of all *P. insidiosum* strains from other species (Figure 1). Moreover, *P. insidiosum* also formed three major clades, corresponding to the rDNA-based genotypes (Table 1), in which clade-I and -II had more similar gene contents than clade-III. A heat map of 100 representative genes showed a firm agreement with the dendrogram-based grouping of the organisms (Figure 1). These results indicate that gene content variation was essential in the evolutionary divergence of *P. insidiosum* from other oomycete species and within the same species.

**Figure 2:**
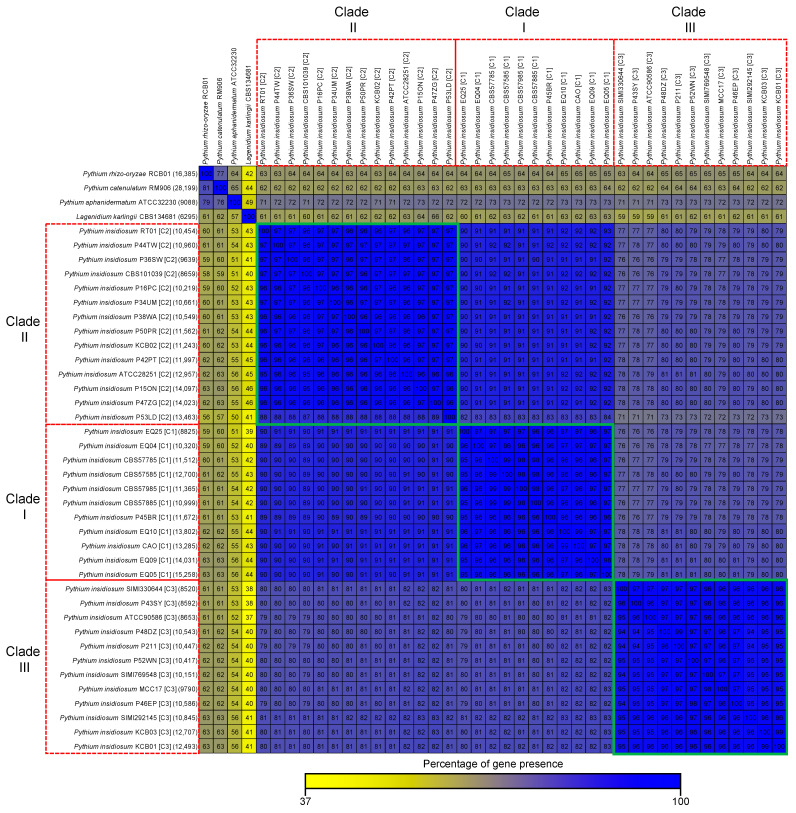
Pairwise gene content comparison of the genes of 37 *P. insidiosum* strains and four other oomycete species. Each parenthesis shows the total number of unique genes in each genome. Each cell shows how many genes the genome on the left has in common with the genome at the top, as a percentage. The red boxes indicate the rDNA-based genotypes (Clade-I, -II, and -III) of *P. insidiosum*. The green boxes show the percentage of shared genes of *P. insidiosum* within the same clade. The color gradience (yellow to blue) reflects the level of gene presence (from 37 to 100%).

To measure the similarity of the gene content of different genomes, we compared all the genes in each genome with all the genes in the other genomes (Figure 2). We calculated the percentages of genes shared between one genome (in each row) and the other genomes (in the columns) to summarize the gene content similarities. *P. insidiosum* strains from the same clade had a high gene content similarity, from 87% to 99% (average: 96%), as seen in Figure 2 (green squares). However, *P. insidiosum* strains from different clades had a lower gene content similarity, from 71 to 93% (average: 83%). The gene content similarity between *P. insidiosum* and other oomycetes was relatively lower, from 37% to 73% (average: 64%), showing that *P. insidiosum* had a different and unique set of genes that might be related to its ability to cause disease in humans and animals.

### 3.4. Core and Variable Genes of P. insidiosum

**Figure 3:**
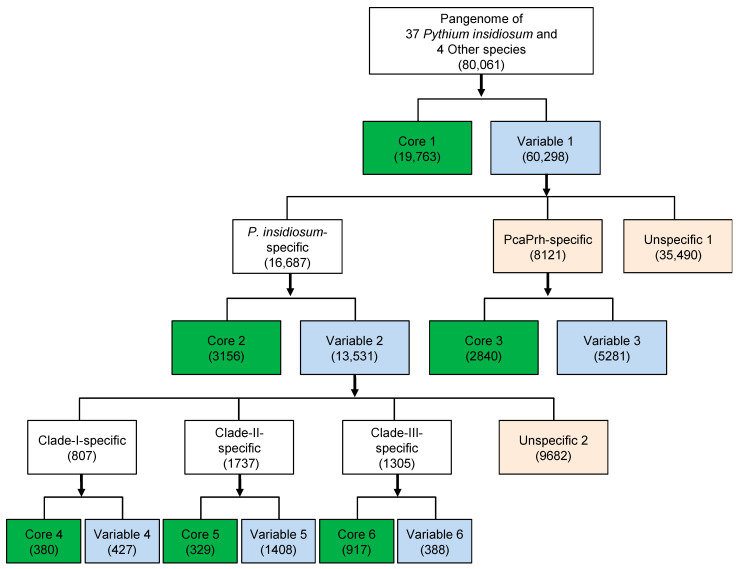
Stepwise classification representing core and variable genes identified in 37 *P. insidiosum* strains and four other oomycetes. The core genes (shared in all genomes) at the genus (Core-1), species (Core-2 for *P. insidiosum* and Core-3 for *P. catenulatum* and *P. rhizo-oryzae* (PcaPrh)), and clade/genotype (Core-4, -5, and -6) levels are shown in green boxes. The variable genes (shared in at least one but not all genomes) at the genus (Variable-1), species (Variable-2 and -3), and clade (Variable-4, -5, and -6) levels are shown in light-blue boxes. Genes that are not unique to *P. insidiosum*, *P. catenulatum,* and *P. rhizo-oryzae* and may be shared with *P. karlingii* and *P. aphanidermatum* are defined as “Unspecific-1”. Non-specific genes shared between two but not all clades of *P. insidiosum* are referred to as “Unspecific-2”.

Unlike most pathogenic oomycetes, which affect plants, *P. insidiosum* infects humans and animals. Data generated here (Table 1; Figure 1 and Figure 2) showed that different strains of *P. insidiosum* had a high degree of genomic variation, which may reflect their evolutionary history and adaptation to different hosts (i.e., humans, horses, dogs) and geographic environments (i.e., Americas, Asia, and Australia). However, to cause an infection in humans and animals, such different *P. insidiosum* strains should contain preserved genes with pathogenic functions distinct from genes that are variably present in the other oomycetes. To investigate this, we grouped 80,061 homologous gene clusters from *P. insidiosum* and other oomycetes into core and variable categories at different levels (i.e., genus, species, and clade/genotype). The genes were divided into Core-1 and Variable-1 (Figure 3). Core-1 genes were considered housekeeping genes (*n* = 19,763) presented in all organisms. Variable-1 genes were accessory genes (*n* = 60,298) that vary among genomes. Variable-1 genes were subdivided into three categories: *P. insidiosum*-specific, PcaPrh-specific, and Unspecific-1. *P. insidiosum*-specific genes (*n* = 16,687) were unique to *P. insidiosum* and not found in other species. PcaPrh-specific genes (*n* = 8121) were present only within the two non-pathogenic *Pythium* species (*P. catenulatum* and *P. rhizo-oryzae*). Unspecific-1 genes (*n* = 35,490) were not unique to *P. insidiosum*, *P. catenulatum,* and *P. rhizo-oryzae* and may be shared with other species, including *P. karlingii* and *P. aphanidermatum*.

The *P. insidiosum*-specific genes can be split into Core-2 and Variable-2. Core-2 genes (*n* = 3156) were common to all *P. insidiosum* genomes, whereas Variable-2 genes (*n* = 13,531) were variable among genomes of this species. Similarly, the PcaPrh-specific genes were subgrouped into Core-3 (*n* = 2840) and Variable-3 (*n* = 5281) genes. The gene classification in *P. insidiosum* and four other oomycetes was summarized in Figure 3. Core-2 genes are significant because some may be responsible for the species-specific virulence required for the pathogenesis of pythiosis in humans and animals. Moreover, Core-2 genes may be potential targets for detecting different *P. insidiosum* strains to improve pythiosis diagnosis and for developing an anti-*P. insidiosum* therapy (i.e., drug and vaccine designs) to promote pythiosis prevention and treatment.

**Figure 4:**
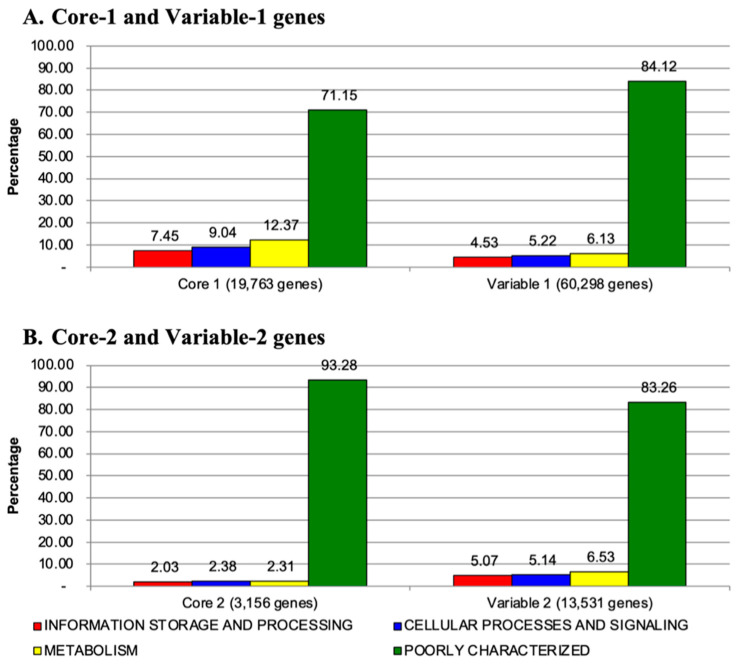
Clusters of Orthologous Groups (COG) of the Core and Variable genes of *P. insidiosum* and other oomycetes. Percentages of the genes assigned to one of the four COG functional categories are shown for (**A**) Core-1 and Variable-1 genes (Pangenome) and (**B**) Core-2 and Variable-2 genes (*P. insidiosum*-specific). According to the COG functional categories, the red bar represents information storage and processing, the blue bar represents cellular processes and signaling, the yellow bar represents metabolism, and the green bar represents poorly characterized or hypothetical proteins.

The COG functional classification [34] was adopted to annotate the Pangenome-level genes (Core-1 and Variable-1; Figure 4A) and *P. insidiosum*-specific genes (Core-2 and Variable-2; Figure 4B). Most Core-1 (71.2%), Variable-1 (84.1%), Core-2 (93.3%), and Variable-2 (83.3%) genes were assigned as genes encoding poorly characterized or hypothetical proteins. The lesser portion of the gene products in each group was functionally involved in either (i) information storage and processing (2.0–7.5%), (ii) cellular processes and signaling (2.4–9.0%), or (iii) metabolism (2.3–12.4%). Overall, the Core-1 genes can be functionally annotated at the highest frequency (28.9%), partly due to the genes in this group being shared among all different organisms and then considered housekeeping genes, which are usually better studied than other types of genes. In contrast, a tiny portion of the Core-2 genes (6.7%) can be annotated, indicating the functions of these *P. insidiosum-specific* genes, potentially involved in the pathogenesis of pythiosis, are largely unknown and require exploration.

### 3.5. Evolutionary Relationship of P. insidiosum and Other Oomycetes

**Figure 5:**
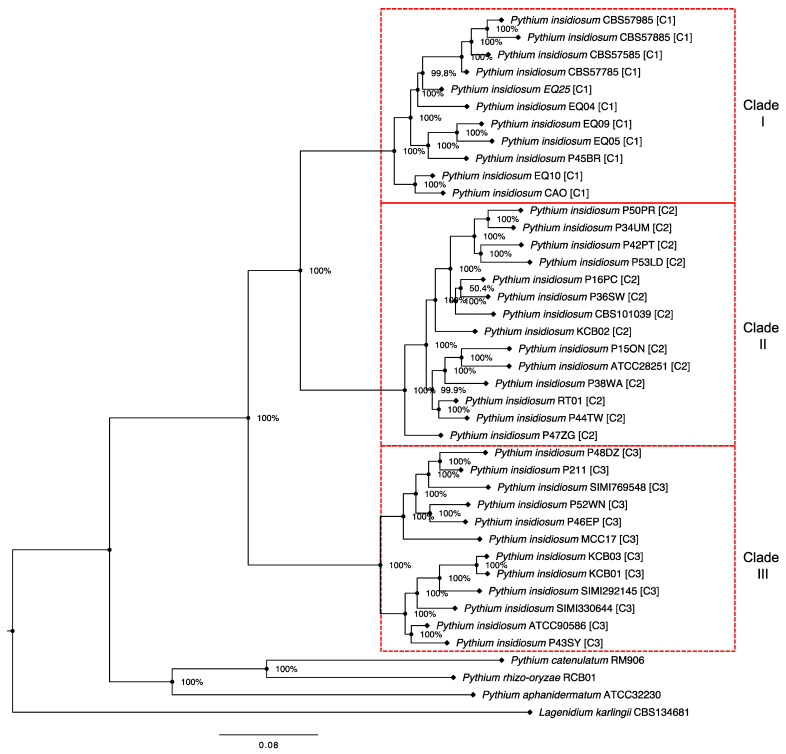
A maximum likelihood-based phylogenetic tree generated from 115 single-copy core genes from 37 *P. insidiosum* and four outgroup oomycete species.

We identified 115 single-copy core genes (a subset of Core-1; Figure 1) in all *P. insidiosum* strains and four other oomycetes. A BLAST search showed that 77 (67%) of these single-copy core genes had known functions, while 38 (33%) were assigned as poorly characterized or hypothetical proteins (Supplementary Table S1). These core genes might be related to the basic features of the oomycetes but not to the mechanisms of infection in humans or animals. These core genes were adopted to investigate the evolutionary relationship between different *P. insidiosum* strains and some closely related oomycetes. We concatenated the 115 genes, aligned them, and removed the gaps, resulting in a 77,751-base-long sequence. A maximum likelihood-based phylogenetic tree was then built from this alignment (Figure 5). The tree showed that *P. insidiosum* strains formed three groups (clade-I, II, and III) distant from other oomycete species. The phylogenetic resolution was high as each *P. insidiosum* strain had its own branch, suggesting they had diverged from a common ancestor.

**Figure 6:**
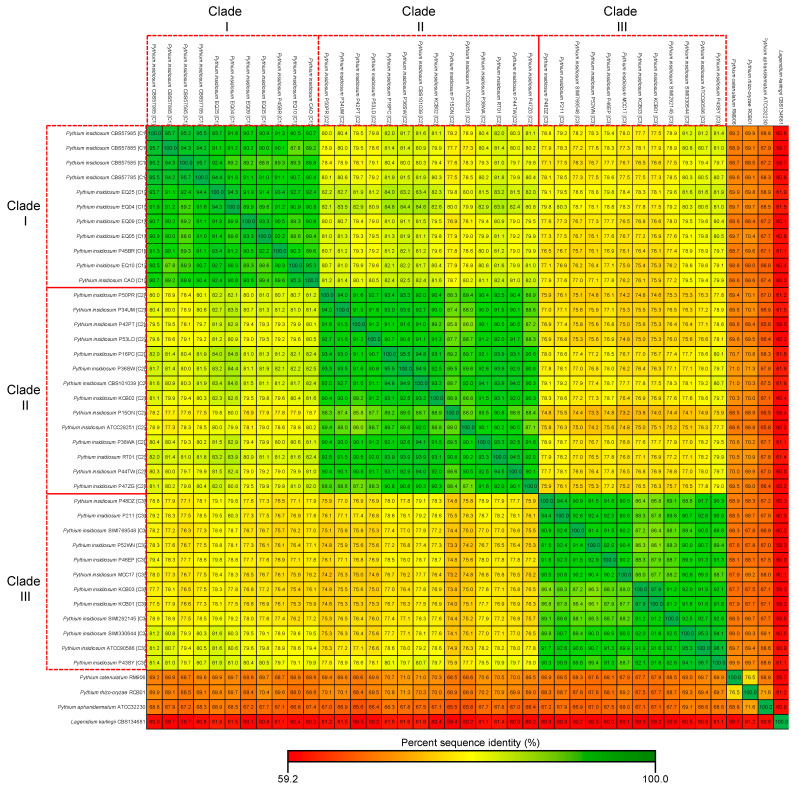
Percent sequence identities of 115 single-copy core-gene sequences calculated between pairs of genomes of 37 *P. insidiosum* strains and four other oomycetes. Red boxes show the *P. insidiosum* strains assigned to rDNA-based genotype clades I, II, and III. Color gradience indicates the degree of sequence identity (%).

As shown in Figure 5, the phylogenetic tree of different *P. insidiosum* strains reveals that the 115 single-copy core gene sequences were more similar within each clade than between different clades. This observation aligned with the percent sequence identities of these core-gene sequences calculated between pairs of genomes of 37 *P. insidiosum* strains and four other oomycetes (Figure 6). For instance, the average percent core-gene sequence identity was 91.4% (range: 88.7–95.7%) within *P. insidiosum* clade-I strains, 91.0% (range: 85.8–95.5%) within clade-II strains, and 90.5% (range: 86.1–97.9%) within clade-III strains. However, when strains from different clades were compared, the average percent of core-gene sequence identity markedly lowered to 78.5% (73.2–84.8%). Furthermore, when *P. insidiosum* was compared with other oomycetes, the average percent sequence identity dropped to only 66.6%. Within *P. insidiosum*, clade-I and -II strains are more closely related to each other than to clade-III strains, suggesting a significant divergence of clade-III from the other strains during the evolutionary history of this pathogenic species.

The distribution of *P. insidiosum* genotypes is strongly linked to geographic location [19,25]. For instance, the Clade-I genotype is exclusively found in American strains, while the isolated Thai strains are always classified as either the Clade-II or -III genotype [19,25]. This study examined the P45BR strain, originally isolated from a dog infected in Thailand. Through hierarchical clustering-based and single-copy core gene-based phylogenomic analyses, we found that this Thai strain is closely related to Clade-I strains from the Americas (Figure 1 and Figure 5). Our finding supports the recent report by Chindampron et al. of another Thai dog infected with a Clade A_th_ (equivalent to Clade-I) strain [40] and confirms that Clade-I strains are already present in Thailand. Regarding the affected hosts, we have observed that Clade-I strains have been found almost exclusively in animals in the Americas, where human cases are rare [1,19,25]. At the same time, humans in Thailand have always been infected with Clade-II and -III strains [1,19,25]. Counting the Clade-I strains isolated from Thai dogs but never found in Thai patients, it is conceivable that Clade-I strains are more specific to animals, while Clade-II and -III strains are more specific to humans. The clade-specific genes of *P. insidiosum* (i.e., Core 4–6 and Variable 4–6; Figure 3) may be responsible for the host-specific virulence.

### 3.6. Exploring P. insidiosum Pathogenicity

A comparative genomic approach, using the Pins Gene Table v2.0, was employed to explore the pathogenicity of *P. insidiosum*. *P. catenulatum* and *P. rhizo-oryzae* inhabit the same aquatic environment as *P. insidiosum* [41] but never appear to cause an infection in humans and animals. Phylogenomic analyses distantly separated *P. catenulatum* and *P. rhizo-oryzae* from *P. insidiosum* (Figure 1 and Figure 4). The genes presented only in *P. catenulatum* or *P. rhizo-oryzae* (assigned in the PcaPrh-specific group; *n* = 8121; Figure 3) were less likely to play a role in an infection in humans and animals. In contrast, the core genes presented in all *P. insidiosum* strains (Core-2 genes; *n* = 3156; Figure 3), but not in the non-pathogenic *Pythium* species, might be centrally involved in the pathogenesis of pythiosis.

To elaborate more on the pathogenicity of *P. insidiosum*, we compared the 3156 Core-2 genes with the MvirDB database containing about 30,000 records of microbial virulence proteins and toxins, each assigned a virulence factor ID (VFID) [35]. We found that 112 (3.5%) of the Core-2 genes had significant matches with 66 VFIDs exhibiting sequence identity ranges from 19.5% to 50.0%, with an average sequence identity of 33.3% (Supplementary Table S2). This result indicates that they are potential virulence factors shared by genetically diverse *P. insidiosum* strains. This finding also suggests that *P. insidiosum* might have adopted an array of different pathogenesis mechanisms from other pathogens via lateral gene transfer events. However, with the low sequence identities relative to known virulent genes from other pathogens, additional investigations are needed before this conclusion can be made. Among the MvirDB database hits, several potential virulence factors were homologous to the reticulocyte binding protein (VFID26643; matched by the gene IDs p-cluster126489, p-cluster200695, p-cluster208509, p-cluster534610, and p-cluster644124), the hemagglutinin/adhesin (VFID7841; matched by p-cluster501203 and p-cluster534263), the venom prothrombin activator (VFID31481; matched by p-cluster496872 and p-cluster707160), and the hemoglobin-binding protein (VFID2575; matched by p-cluster474142). These highlighted virulence factors might conceivably link to a pathological feature of pythiosis where infection occurs in arteries, leading to blood clot formation and arterial occlusion [5,15]. Further experiments are required to validate their biological function involved in the pathogenesis of pythiosis.

## 4. Conclusions

An online database called Pins Gene Table v2.0 was developed to facilitate the genomic analysis of *P. insidiosum*. The MGI-based next-generation sequencing technology was used to generate genomic data for 37 genetically diverse strains of *P. insidiosum* isolated from different hosts and geographic locations and four other oomycetes species. The database contains 732,686 genes, which can be grouped into 80,061 unique homologous gene clusters. These gene clusters were categorized into core and variable categories at different levels (i.e., genus, species, and clade/genotype). A high-resolution phylogenomic relationship among *P. insidiosum* strains and other oomycetes was projected through hierarchical clustering and core gene analyses. We identified 3156 *P. insidiosum*-specific genes that were shared among all genotypes/clades and may be responsible for causing disease in humans and animals. After comparing these species-specific genes against the MvirDB database, 112 had significant matches with 66 known virulence proteins, some of which (i.e., reticulocyte-binding protein, hemagglutinin/adhesin, prothrombin activator, and hemoglobin-binding protein) might be involved in blood clot formation and arterial occlusion, a pathological feature of pythiosis. The correlation of genotypes, geographic origins, and affected hosts suggests that clade-I strains are more specific to animals, while clade-II/III strains are more specific to humans. The clade-specific genes might link to host preference. In summary, Pins Gene Table v2.0 is a comprehensive genome database that is easily accessible for users with minimal bioinformatics experience to navigate, compare, and analyze the genomes of this pathogen.

## Figures and Tables

**Table 1:** Genome summary of 37 *P. insidiosum* strains and four related oomycete species (i.e., *P.* catenulatum, *P. rhizo*-oryzae, *P. aphanidermatum*, and *P. karlingii*) used in this study.

Microorganism and Strain [rDNA-Based Genotypes: Clade I, II, or III]	Isolation Source	Country of Origin	Total Number of Contigs	Total Length	GC Content (%)	No. of CDS	Total Coding Sequence Length	Average CDS Size (bp)	Coding Density (%)	N50	Accession Number
*Pythium insidiosum* CBS57985 [I]	Horse	Costa Rica	58,059	56,435,402	57.2	16,248	28,505,008	1754	51	30,031	JANKNM000000000
*Pythium insidiosum* CBS57885 [I]	Horse	Costa Rica	53,226	54,714,377	57.2	15,888	28,206,665	1775	52	31,836	JANKNN000000000
*Pythium insidiosum* CBS57585 [I]	Horse	Costa Rica	102,302	65,770,141	57.0	17,795	29,816,459	1676	45	12,088	JANSWB000000000
*Pythium insidiosum* CBS57785 [I]	Horse	Costa Rica	87,425	61,450,997	56.9	16,619	28,976,299	1744	47	21,234	JANKNL000000000
*Pythium insidiosum* EQ25 [I]	Horse	Brazil	26,171	42,682,656	57.4	12,975	25,446,245	1961	60	58,186	JANKNG000000000
*Pythium insidiosum* EQ04 [I]	Horse	Brazil	42,915	52,351,402	56.9	15,003	27,426,329	1828	52	36,350	JANKNK000000000
*Pythium insidiosum* EQ09 [I]	Horse	Brazil	71,506	66,735,985	57.2	20,106	32,490,626	1616	49	10,672	JANKNI000000000
*Pythium insidiosum* EQ05 [I]	Horse	Brazil	96,117	74,060,062	57.2	21,613	33,277,973	1540	45	5653	JANKNJ000000000
*Pythium insidiosum* P45BR [I]	Dog	Thailand	92,974	62,902,336	56.9	16,833	29,114,310	1730	46	16,663	JANKNE000000000
*Pythium insidiosum* EQ10 [I]	Horse	Brazil	78,558	65,289,697	57.3	19,485	31,040,556	1593	48	11,249	JANKNH000000000
*Pythium insidiosum* CAO [I]	Dog	Brazil	77,829	63,569,142	57.3	18,664	30,460,447	1632	48	15,548	JANKNF000000000
*Pythium insidiosum* P50PR [II]	Human	Thailand	51,305	53,992,893	57.5	16,110	27,473,875	1705	51	45,810	JANKNV000000000
*Pythium insidiosum* P34UM [II]	Human	Thailand	55,434	52,629,044	57.3	15,109	26,948,508	1784	51	53,527	JANKNB000000000
*Pythium insidiosum* P42PT [II]	Horse	Thailand	73,208	59,704,421	57.2	16,714	28,106,907	1682	47	24,294	JAMFLR000000000
*Pythium insidiosum* P53LD [II]	Human	Thailand	86,880	67,309,556	57.3	18,359	31,415,000	1711	47	34,658	JANSWC000000000
*Pythium insidiosum* P16PC [II]	Human	Thailand	49,078	51,152,361	57.4	14,646	26,529,743	1811	52	56,597	JANKNC000000000
*Pythium insidiosum* P36SW [II]	Human	Thailand	42,097	48,130,377	57.3	13,934	26,063,402	1870	54	66,694	JANKNA000000000
*Pythium insidiosum* CBS101039 [II]	Human	India	35,148	44,055,376	57.4	12,707	24,941,028	1963	57	80,790	JANKNQ000000000
*Pythium insidiosum* KCB02 [II]	Environment	Thailand	64,641	56,729,906	57.1	15,850	27,282,935	1721	48	38,164	JANKNU000000000
*Pythium insidiosum* P15ON [II]	Human	Thailand	67,980	64,599,045	57.4	20,033	31,681,612	1581	49	10,060	JANKND000000000
*Pythium insidiosum* ATCC28251 [II]	Horse	New Guinea	48,464	58,405,737	57.5	18,340	30,143,400	1644	52	26,790	JANKNR000000000
*Pythium insidiosum* P38WA [II]	Human	Thailand	44,071	51,344,795	57.2	15,094	26,749,622	1772	52	58,072	JANKNP000000000
*Pythium insidiosum* RT01 [II]	Environment	Thailand	39,862	49,900,667	57.4	14,882	26,406,073	1774	53	60,023	JANKNT000000000
*Pythium insidiosum* P44TW [II]	Human	Thailand	43,956	50,906,565	57.6	15,366	26,833,944	1746	53	57,463	JANKNO000000000
*Pythium insidiosum* P47ZG [II]	Horse	Thailand	86,960	69,697,082	57.1	19,687	30,685,124	1559	44	5672	JAMFLQ000000000
*Pythium insidiosum* P48DZ [III]	Environment	Thailand	38,467	74,633,896	57.6	21,938	44,167,578	2013	59	48,527	JAMFLP000000000
*Pythium insidiosum* P211 [III]	Human	Thailand	37,700	73,313,598	57.7	21,451	43,310,684	2019	59	46,352	JANFOR000000000
*Pythium insidiosum* SIMI769548 [III]	Human	Thailand	39,378	68,593,535	57.6	19,930	40,231,227	2019	59	47,983	JANFOV000000000
*Pythium insidiosum* P52WN [III]	Human	Thailand	39,964	75,427,560	57.5	22,015	44,220,684	2009	59	48,661	JANFOS000000000
*Pythium insidiosum* P46EP [III]	Human	Thailand	36,214	74,147,894	57.5	21,803	43,565,929	1998	59	47,341	JANFOQ000000000
*Pythium insidiosum* MCC17 [III]	Human	Thailand	64,505	66,914,522	57.6	18,215	36,586,559	2009	55	44,304	JANKNS000000000
*Pythium insidiosum* KCB03 [III]	Environment	Thailand	84,399	67,876,891	57.6	17,345	29,762,456	1716	44	12,606	JANFOT000000000
*Pythium insidiosum* KCB01 [III]	Environment	Thailand	74,224	66,027,843	57.7	17,185	29,574,447	1721	45	14,844	JANFOU000000000
*Pythium insidiosum* SIMI292145 [III]	Human	Thailand	66,261	57,978,219	57.6	14,890	26,996,604	1813	47	23,734	JANFOW000000000
*Pythium insidiosum* SIMI330644 [III]	Human	Thailand	58,297	47,650,386	57.7	12,008	23,818,272	1984	50	33,153	JANFOX000000000
*Pythium insidiosum* ATCC90586 [III]	Human	USA	47,461	47,712,244	57.5	12,249	24,368,249	1989	51	38,574	JAKCXL000000000
*Pythium insidiosum* P43SY [III]	Human	Thailand	40,285	46,101,683	57.4	12,146	23,878,545	1966	52	40,098	JAKCXM000000000
*Pythium catenulatum* RM906	Environment	Thailand	119,153	98,985,705	61.5	47,975	44,233,641	922	45	1526	JAMFLM000000000
*Pythium rhizo-oryzae* RCB01	Environment	Thailand	36,255	95,734,336	59.8	35,827	60,762,112	1696	63	17,880	JAMFLL000000000
*Pythium aphanidermatum* ATCC32230	Plant	USA	68,387	59,209,978	53.3	12,160	18,205,062	1497	31	2799	JAMFLN000000000
*Paralagenidium karlingii* CBS134681	Dog	USA	65,760	57,394,197	51.2	7489	18,899,676	2524	33	40,792	JAMFLO000000000

## Data Availability

The draft genome sequences of 37 *P. insidiosum* strains and four other oomycete species are retrievable from the DDBJ/NCBI databases through the accession numbers shown in Table 1.

## Supplementary Materials

The following supporting information can be downloaded at: https://www.mdpi.com/article/10.3390/jof10020112/s1, Table S1. 115 single-copy core genes found across the genomes of 37 *P. insidiosum* and 4 related oomycetes. Table S2. 112 *P. insidiosum*-specific (Core-2) genes that matched microbial virulence proteins and toxins deposited in the MvirDB database.
