# Supplementary material for: Pins Gene Table v2.0: An Online Genome Database of 37 Pythium insidiosum Strains for Gene Content Exploration and Phylogenomic Analysis
Source: J Fungi (Basel). 2024 Jan 29;10(2):112. doi: 10.3390/jof10020112 (PMC10889951; doi:10.3390/jof10020112)
Supplement: Supplementary file 1 [file jof-10-00112-s001.zip › jof-2749567-supplementary.pdf]

**Supplementary Table S1.** 115 single-copy core genes found across the genomes of 37 *P. insidiosum* and 4 related oomycetes.

| Gene cluster ID | Gene length (bp) | Gene product description |
|---|---|---|
| p-cluster594855 | 10837 | Elongation factor 3, putative |
| p-cluster682933 | 10071 | ABC transporter lipid export ABC Family |
| p-cluster312751* | 7015 | Hypothetical protein |
| p-cluster539815 | 5833 | Pleiotropic drug resistance protein ABC superfamily |
| p-cluster454010 | 5716 | Calcium-translocating p-type ATPase, pmca-type |
| p-cluster510208* | 5687 | Hypothetical protein |
| p-cluster099966 | 5560 | Phospholipid-transporting ATPase, putative, partial |
| p-cluster597669* | 5463 | Hypothetical protein |
| p-cluster412135 | 5305 | Glucose transporter |
| p-cluster095503 | 5168 | Myosin-like protein |
| p-cluster421014 | 4840 | Intraflagellar transport protein 122 |
| p-cluster361620 | 4820 | Structural maintenance of chromosomes protein |
| p-cluster597184 | 4683 | Arf-gap with GTPase, ank repeat and ph domain-containing protein 9 |
| p-cluster306513 | 4594 | Transmembrane protein |
| p-cluster316612 | 4496 | Elicitor-like transglutaminase |
| p-cluster141083 | 4319 | Glycolate oxidase, subunit glcd, partial |
| p-cluster477503* | 4192 | Hypothetical protein |
| p-cluster464758 | 4049 | Exportin-2-like protein |
| p-cluster449940* | 3978 | Hypothetical protein |
| p-cluster193710* | 3912 | Hypothetical protein |
| p-cluster532599 | 3869 | Periodic tryptophan protein 2 |
| p-cluster065379* | 3730 | Hypothetical protein |
| p-cluster434954 | 3539 | Dolichyl-diphosphooligosaccharide-protein glycosylltransferase subunit |
| p-cluster008242 | 3525 | Luminal-binding protein |
| p-cluster644157* | 3499 | Hypothetical protein |
| p-cluster359933* | 3432 | Hypothetical protein |
| p-cluster709453 | 3418 | Long-chain-fatty-acid-CoA ligase |
| p-cluster464826 | 3304 | Pre-rRNA-processing protein tsr1 |
| p-cluster709189 | 3232 | Peroxisome assembly factor |
| p-cluster196014* | 3143 | Hypothetical protein |
| p-cluster356030* | 3049 | Hypothetical protein |
| p-cluster432958* | 3049 | Hypothetical protein |
| p-cluster357833* | 2988 | Hypothetical protein |
| p-cluster536025 | 2948 | Polypyrimidine tract-binding protein |
| p-cluster054118 | 2947 | Calcineurin-like phosphoesterase |
| p-cluster194771* | 2945 | Hypothetical protein |
| p-cluster662691 | 2844 | ABC transporter-like protein, partial |
| p-cluster554166 | 2765 | Glucose transporter |
| p-cluster189931* | 2723 | Hypothetical protein |
| p-cluster458608* | 2720 | Hypothetical protein |
| p-cluster055747* | 2685 | Hypothetical protein |
| p-cluster575357 | 2643 | Adenylate cyclase, putative |
| p-cluster579716 | 2639 | Tigger transposable element-derived protein 6 |
| p-cluster082612 | 2610 | 2-isopropylmalate synthase |
| p-cluster729567* | 2603 | Hypothetical protein |
| p-cluster386600 | 2559 | Threonine-tRNA ligase |
| p-cluster540880 | 2503 | Lysosomal pro-x carboxypeptidase |
| p-cluster054458 | 2422 | Radial spoke head 10 family protein |
| p-cluster472248 | 2393 | Cytosol aminopeptidase |
| p-cluster361606 | 2314 | 2,4-dienoyl-coa reductase |
| p-cluster071300 | 2274 | Translocation protein sec63 |
| p-cluster524538 | 2271 | T-complex protein 1, theta subunit |
| p-cluster476051* | 2270 | Hypothetical protein |
| p-cluster324713* | 2160 | Hypothetical protein |
| p-cluster590582 | 2143 | Exopolysaccharide phosphotransferase |
| p-cluster197205 | 2135 | Glucose-6-phosphate isomerase |
| p-cluster362721* | 2123 | Hypothetical protein |
| p-cluster092525 | 2093 | Fact complex subunit ssrp1 |
| p-cluster198537* | 2055 | Hypothetical protein |
| p-cluster190165 | 2039 | Cystathionine beta-lyase |

| | | |
|---|---|---|
| p-cluster556727 | 2037 | Phosphoglycerate kinase |
| p-cluster476199 | 2018 | ATP-dependent protease hslvu, ATPase subunit |
| p-cluster474688 | 1955 | Aldehyde dehydrogenase |
| p-cluster397427 | 1954 | Actin-like protein |
| p-cluster458607 | 1947 | Cyclin-y-like protein, partial |
| p-cluster238230 | 1917 | Acyl-CoA dehydrogenase family member 9, mitochondrial precursor |
| p-cluster331766* | 1910 | Hypothetical protein |
| p-cluster508816 | 1888 | Dihydrolipoyl dehydrogenase 1, mitochondrial precursor |
| p-cluster190599* | 1886 | Hypothetical protein |
| p-cluster160158 | 1858 | 6-phosphogluconate dehydrogenase |
| p-cluster125186 | 1842 | Pyruvate dehydrogenase e1 component subunit beta, mitochondrial |
| p-cluster227751 | 1791 | Glutathione-dependent formaldehyde dehydrogenase |
| p-cluster190378 | 1773 | Pyruvate dehydrogenase e1 alpha subunit |
| p-cluster055980 | 1761 | Rna 3'-terminal phosphate cyclase |
| p-cluster072639 | 1731 | Aminotransferase |
| p-cluster408676* | 1697 | Hypothetical protein |
| p-cluster309873* | 1575 | Hypothetical protein |
| p-cluster578941 | 1568 | 60s ribosomal protein |
| p-cluster646099 | 1555 | Serine/threonine-protein phosphatase ppe1 |
| p-cluster539607 | 1551 | Asparagine-trna ligase |
| p-cluster598164 | 1525 | Enoyl-coa hydratase, mitochondrial precursor |
| p-cluster063940 | 1523 | Protein phosphatase 2c |
| p-cluster196735 | 1480 | Bifunctional fold protein |
| p-cluster617622 | 1428 | Phosphoserine phosphatase |
| p-cluster012729 | 1398 | S-adenosylmethionine synthase 2 |
| p-cluster423633* | 1371 | Hypothetical protein |
| p-cluster192633 | 1361 | Acyl-CoA desaturase |
| p-cluster412337 | 1361 | Sporangia induced 28 kDa inner dynein arm light chain |
| p-cluster676979 | 1359 | Purine nucleoside phosphorylase i, inosine and guanosine-specific |
| p-cluster349235 | 1357 | 14-3-3 protein epsilon |
| p-cluster083401 | 1247 | Voltage-dependent anion-selective channel protein |
| p-cluster556722* | 1237 | Hypothetical protein |
| p-cluster149186* | 1235 | Hypothetical protein |
| p-cluster173431 | 1212 | Myosin-like protein |
| p-cluster053234* | 1110 | Hypothetical protein |
| p-cluster000460 | 1106 | Vesicle-associated membrane protein, putative, partial |
| p-cluster410772* | 1068 | Hypothetical protein |
| p-cluster474440* | 1060 | Hypothetical protein |
| p-cluster453101 | 1040 | Putative methyltransferase |
| p-cluster476353* | 904 | Hypothetical protein |
| p-cluster452444* | 768 | Hypothetical protein |
| p-cluster263392 | 741 | Gtp-binding protein parf |
| p-cluster450789* | 732 | Hypothetical protein |
| p-cluster124244* | 685 | Hypothetical protein |
| p-cluster270302 | 679 | Translation elongation factor if5a |
| p-cluster193243 | 549 | Ribosomal protein l23 |
| p-cluster452513 | 547 | Eukaryotic translation initiation factor 1b |
| p-cluster452369 | 435 | Ribosomal protein l11 |
| p-cluster044680 | 429 | Mitochondrial/chloroplast ribosomal protein l13 |
| p-cluster452752 | 398 | 60s ribosomal protein l44 |
| p-cluster236498* | 390 | Hypothetical protein |
| p-cluster012383 | 387 | 13 kDa ribonucleoprotein-associated protein |
| p-cluster261767* | 383 | Hypothetical protein |
| p-cluster005452 | 351 | Cytochrome c |
| p-cluster056982 | 285 | U6 snrnaassociated smlike protein lsm3 |

**Footnote:**
*Hypothetical protein

**Supplementary Table S2.** 112 *P. insidiosum*-specific (Core-2) genes that matched microbial virulence proteins and toxins deposited in the MvirDB database.

| Query Gene ID | Hit VFID | Hit Gene Description [Microorganism] | Query Gene Length (bp) | Hit Gene Length (bp) | Alignment Identify (%) | Query Length Alignment Coverage (%) |
|---|---|---|---|---|---|---|
| p-cluster695607 | 2428 | 30K adhesin-related protein [Mycoplasma pneumoniae] | 296 | 274 | 47.7 | 29.1 |
| p-cluster397479 | 2478 | Mycocerosic acid synthase [Mycobacterium tuberculosis] | 772 | 2111 | 33.1 | 20.3 |
| p-cluster474142 | 2575 | Hemoglobin-binding protein [Haemophilus influenzae] | 1043 | 1084 | 32.9 | 7.3 |
| p-cluster538005 | 2911 | Phosphatase | 737 | 171 | 41.5 | 7.2 |
| p-cluster087850 | 4404 | Alpha-latroinsectotoxin | 490 | 1411 | 31.8 | 25.9 |
| p-cluster410852 | 4404 | Alpha-latroinsectotoxin | 564 | 1411 | 26.3 | 25.7 |
| p-cluster712810 | 4404 | Alpha-latroinsectotoxin-Lt1a | 143 | 1411 | 28.9 | 79.7 |
| p-cluster337533 | 5926 | Tryptophan repeat gene family protein | 264 | 297 | 48.8 | 16.3 |
| p-cluster376328 | 6974 | Cyclin D homolog | 283 | 254 | 32.0 | 61.5 |
| p-cluster146673 | 7247 | 2,3-dihydroxybenzoate-2,3-dehydrogenase [Shigella flexneri] | 453 | 248 | 26.2 | 38.6 |
| p-cluster709624 | 7458 | Chaperone DnAJ [Bacteroides thetaiotaomicron] | 247 | 321 | 30.7 | 55.1 |
| p-cluster608871 | 7464 | Oxidoreductase [Bacteroides thetaiotaomicron] | 717 | 245 | 28.3 | 24.7 |
| p-cluster501203 | 7841 | Filamentous hemagglutinin/adhesin [Bordetella pertussis] | 288 | 3590 | 29.8 | 34.4 |
| p-cluster534263 | 7841 | Filamentous hemagglutinin/adhesin [Bordetella pertussis] | 672 | 3590 | 34.0 | 27.2 |
| p-cluster404363 | 7938 | Pneumococcal surface protein A [Streptococcus pneumoniae] | 181 | 744 | 43.3 | 32.6 |
| p-cluster187984 | 7988 | Phosphatidate cytidylyltransferase [Enterococcus faecalis] | 477 | 266 | 42.3 | 26.2 |
| p-cluster495776 | 8167 | Internalin A [Listeria monocytogenes] | 1149 | 800 | 33.3 | 11.0 |
| p-cluster721089 | 8434 | Hypothetical protein lpg2490 [Legionella pneumophila] | 5018 | 1294 | 22.0 | 11.3 |
| p-cluster574646 | 9543 | Zinc finger and BTB domain-containing protein 7C | 739 | 619 | 32.7 | 18.9 |
| p-cluster578127 | 9543 | Zinc finger and BTB domain-containing protein 7C | 1019 | 619 | 34.9 | 10.7 |
| p-cluster199836 | 10373 | 2,3-dihydro-2,3-dihydroxybenzoate dehydrogenase | 236 | 261 | 33.3 | 39.4 |
| p-cluster322171 | 13372 | Pyruvate kinase [Salmonella typhimurium] | 151 | 470 | 46.2 | 60.3 |
| p-cluster357085 | 13763 | Helicase [Vibrio cholerae] | 1242 | 940 | 24.0 | 23.7 |
| p-cluster706532 | 14026 | Lysil-tRNA synthetase LysU [Escherichia coli] | 700 | 498 | 38.5 | 67.1 |
| p-cluster187093 | 14152 | Serine/threonine protein kinase PpkA [Pseudomonas aeruginosa] | 710 | 1032 | 32.4 | 31.1 |
| p-cluster460671 | 14152 | Serine/threonine protein kinase PpkA [Pseudomonas aeruginosa] | 849 | 1032 | 30.6 | 25.1 |
| p-cluster534559 | 14152 | Serine/threonine protein kinase PpkA [Pseudomonas aeruginosa] | 1378 | 1032 | 27.7 | 16.1 |
| p-cluster566997 | 14152 | Serine/threonine protein kinase PpkA [Pseudomonas aeruginosa] | 485 | 1032 | 32.2 | 36.5 |
| p-cluster598855 | 14152 | Serine/threonine protein kinase PpkA [Pseudomonas aeruginosa] | 923 | 1032 | 27.7 | 30.8 |
| p-cluster298495 | 14529 | Astacin-like metalloprotease toxin | 1593 | 264 | 25.6 | 13.5 |
| p-cluster475982 | 15449 | Hypothetical protein bll8085 [Bradyrhizobium japonicum] | 548 | 669 | 30.4 | 20.1 |
| p-cluster507512 | 15953 | Hypothetical protein BT_2630 [Bacteroides thetaiotaomicron] | 763 | 316 | 28.0 | 29.1 |
| p-cluster356795 | 16229 | Hypothetical protein [Escherichia coli] | 1108 | 504 | 30.9 | 13.3 |
| p-cluster570708 | 16240 | Transacylase [Escherichia coli] | 630 | 422 | 27.9 | 45.2 |
| p-cluster694284 | 16244 | 3-hydroxyacyl-CoA dehydrogenase [Escherichia coli] | 330 | 289 | 28.2 | 51.8 |
| p-cluster348793 | 16625 | Ornithine carbamoyltransferase 2 [Escherichia coli] | 1278 | 334 | 37.1 | 21.5 |
| p-cluster572952 | 16625 | Ornithine carbamoyltransferase 2 [Escherichia coli] | 140 | 334 | 32.6 | 67.9 |

| | | | | | | |
|---|---|---|---|---|---|---|
| p-cluster546828 | 17975 | Ortho-methyltransferase [Mesorhizobium loti] | 176 | 344 | 28.8 | 79.0 |
| p-cluster394933 | 18009 | Hypothetical protein mlr8090 [Mesorhizobium loti] | 1531 | 274 | 33.3 | 12.9 |
| p-cluster099653 | 18169 | WD-40 repeat-containing protein [Nostoc sp.] | 715 | 1526 | 34.0 | 20.4 |
| p-cluster104690 | 18169 | WD-40 repeat-containing protein [Nostoc sp.] | 1012 | 1526 | 24.3 | 27.7 |
| p-cluster460953 | 18169 | WD-40 repeat-containing protein [Nostoc sp.] | 768 | 1526 | 31.3 | 37.0 |
| p-cluster509407 | 18169 | WD-40 repeat-containing protein [Nostoc sp.] | 729 | 1526 | 26.8 | 29.2 |
| p-cluster628984 | 18169 | WD-40 repeat-containing protein [Nostoc sp.] | 478 | 1526 | 28.6 | 74.1 |
| p-cluster651543 | 18169 | WD-40 repeat-containing protein [Nostoc sp.] | 962 | 1526 | 26.6 | 32.0 |
| p-cluster278739 | 18492 | tRNA pseudouridine synthase A [Streptococcus agalactiae] | 641 | 258 | 31.5 | 31.7 |
| p-cluster051977 | 18783 | Phosphomethylpyrimidine kinase [Streptococcus agalactiae] | 1008 | 252 | 26.2 | 27.0 |
| p-cluster321888 | 18794 | Pyridine nucleotide-disulfide oxidoreductase [Streptococcus agalactiae] | 305 | 439 | 30.3 | 65.9 |
| p-cluster271256 | 18887 | Hypothetical protein gbs0204 [Streptococcus agalactiae] | 327 | 249 | 29.8 | 71.9 |
| p-cluster153820 | 19086 | Oxidoreductase [Streptomyces coelicolor] | 128 | 259 | 29.4 | 93.0 |
| p-cluster508567 | 19086 | Oxidoreductase [Streptomyces coelicolor] | 1256 | 259 | 31.8 | 11.7 |
| p-cluster520923 | 19524 | AAA family ATPase [Shewanella oneidensis] | 785 | 680 | 28.2 | 19.6 |
| p-cluster366095 | 20413 | Sialic acid transporter [Salmonella enterica] | 190 | 426 | 36.6 | 58.9 |
| p-cluster589962 | 20551 | Metallothionein SmtA [Salmonella enterica] | 307 | 267 | 29.5 | 44.6 |
| p-cluster139276 | 20945 | Oxidoreductase [Xylella fastidiosa] | 887 | 247 | 29.7 | 20.9 |
| p-cluster132374 | 23568 | Dimethyltransferase [Photorhabdus luminescens] | 637 | 272 | 32.9 | 36.7 |
| p-cluster556115 | 25250 | TetT [Enterococcus faecalis] | 850 | 651 | 27.0 | 21.2 |
| p-cluster299054 | 25899 | CRISP-PHI1 [Philodryas olfersii] | 910 | 232 | 31.5 | 18.5 |
| p-cluster444101 | 25936 | Alpha-latrotoxin precursor [Latrodectus tredecimguttatus] | 68 | 1401 | 36.7 | 88.2 |
| p-cluster217227 | 26380 | ATP-binding cassette protein [Cryptosporidium parvum] | 843 | 1431 | 44.6 | 10.9 |
| p-cluster282246 | 26380 | ATP-binding cassette protein [Cryptosporidium parvum] | 2290 | 1431 | 23.2 | 13.8 |
| p-cluster051071 | 26400 | cGMP dependent protein kinase [Toxoplasma gondii] | 1115 | 994 | 34.6 | 18.2 |
| p-cluster098355 | 26400 | cGMP dependent protein kinase [Toxoplasma gondii] | 461 | 994 | 35.6 | 35.1 |
| p-cluster160648 | 26400 | cGMP dependent protein kinase [Toxoplasma gondii] | 796 | 994 | 32.9 | 24.2 |
| p-cluster320677 | 26400 | cGMP dependent protein kinase [Toxoplasma gondii] | 473 | 994 | 31.5 | 34.7 |
| p-cluster456679 | 26400 | cGMP dependent protein kinase [Toxoplasma gondii] | 1377 | 994 | 38.6 | 16.9 |
| p-cluster461715 | 26400 | cGMP dependent protein kinase [Toxoplasma gondii] | 479 | 994 | 29.2 | 59.5 |
| p-cluster510105 | 26400 | cGMP dependent protein kinase [Toxoplasma gondii] | 1707 | 994 | 34.3 | 17.4 |
| p-cluster526470 | 26400 | cGMP dependent protein kinase [Toxoplasma gondii] | 1069 | 994 | 33.1 | 12.1 |
| p-cluster615580 | 26400 | cGMP dependent protein kinase [Toxoplasma gondii] | 1136 | 994 | 31.2 | 16.3 |
| p-cluster643963 | 26400 | cGMP dependent protein kinase [Toxoplasma gondii] | 412 | 994 | 37.1 | 28.2 |
| p-cluster697518 | 26400 | cGMP dependent protein kinase [Toxoplasma gondii] | 2273 | 994 | 26.8 | 11.2 |
| p-cluster605319 | 26433 | Regulator of chromosome condensation 1 [Toxoplasma gondii] | 774 | 1155 | 31.4 | 25.8 |
| p-cluster117786 | 26467 | Aspartyl proteinase eimepsin [Eimeria tenella] | 303 | 468 | 34.6 | 42.9 |
| p-cluster191433 | 26470 | cGMP-dependent protein kinase [Eimeria tenella] | 143 | 1003 | 32.8 | 94.4 |
| p-cluster197376 | 26470 | cGMP-dependent protein kinase [Eimeria tenella] | 785 | 1003 | 41.5 | 36.4 |
| p-cluster285589 | 26470 | cGMP-dependent protein kinase [Eimeria tenella] | 543 | 1003 | 31.0 | 40.0 |
| p-cluster308454 | 26470 | cGMP-dependent protein kinase [Eimeria tenella] | 808 | 1003 | 37.1 | 34.5 |
| p-cluster446407 | 26470 | cGMP-dependent protein kinase [Eimeria tenella] | 595 | 1003 | 43.0 | 13.3 |

| | | | | | | |
|---|---|---|---|---|---|---|
| p-cluster460500 | 26470 | cGMP-dependent protein kinase [Eimeria tenella] | 830 | 1003 | 36.8 | 24.1 |
| p-cluster503872 | 26470 | cGMP-dependent protein kinase [Eimeria tenella] | 504 | 1003 | 31.0 | 43.7 |
| p-cluster058799 | 26605 | CTRP [Plasmodium falciparum] | 244 | 2114 | 40.9 | 25.4 |
| p-cluster318682 | 26605 | CTRP [Plasmodium falciparum] | 419 | 2114 | 44.1 | 13.6 |
| p-cluster543319 | 26605 | CTRP [Plasmodium falciparum] | 176 | 2114 | 36.7 | 27.8 |
| p-cluster200695 | 26643 | Reticulocyte binding protein 2 homolog a [Plasmodium falciparum] | 984 | 3130 | 19.5 | 22.0 |
| p-cluster208509 | 26643 | Reticulocyte binding protein 2 homolog a [Plasmodium falciparum] | 607 | 3130 | 32.1 | 21.9 |
| p-cluster126489 | 26643 | Reticulocyte binding protein 2 homolog a [Plasmodium falciparum] | 2366 | 3130 | 36.5 | 6.9 |
| p-cluster534610 | 26643 | Reticulocyte binding protein 2 homolog a [Plasmodium falciparum] | 495 | 3130 | 33.3 | 20.8 |
| p-cluster644124 | 26643 | Reticulocyte binding protein 2 homolog a [Plasmodium falciparum] | 658 | 3130 | 29.4 | 21.0 |
| p-cluster139183 | 26985 | Adhesin protein AP51-3 [Trichomonas vaginalis] | 110 | 405 | 46.8 | 70.9 |
| p-cluster591236 | 26985 | Adhesin protein AP51-3 [Trichomonas vaginalis] | 131 | 405 | 50.0 | 80.2 |
| p-cluster721070 | 27018 | LPG2 [Leishmania donovani] | 435 | 341 | 29.8 | 73.8 |
| p-cluster538689 | 27170 | Cathepsin B-like cysteine protease [Trypanosoma brucei] | 1742 | 340 | 32.0 | 15.1 |
| p-cluster098089 | 27222 | Calcineurin B subunit [Trypanosoma cruzi] | 742 | 176 | 29.9 | 16.8 |
| p-cluster173714 | 27247 | Trans-sialidase [Trypanosoma cruzi] | 1638 | 1446 | 29.3 | 32.0 |
| p-cluster688261 | 27940 | Trans-sialidase [Trypanosoma cruzi] | 182 | 373 | 44.5 | 60.4 |
| p-cluster060553 | 27997 | Small GTPase Rab5 [Entamoeba histolytica] | 123 | 195 | 38.7 | 61.0 |
| p-cluster542461 | 27997 | Small GTPase Rab5 [Entamoeba histolytica] | 332 | 195 | 32.0 | 46.1 |
| p-cluster563931 | 28000 | Small GTPase Rab11B [Entamoeba histolytica] | 187 | 211 | 43.1 | 38.5 |
| p-cluster621238 | 28000 | Small GTPase Rab11B [Entamoeba histolytica] | 295 | 211 | 41.1 | 59.0 |
| p-cluster681597 | 28000 | Small GTPase Rab11B [Entamoeba histolytica] | 779 | 211 | 38.2 | 25.5 |
| p-cluster457390 | 28063 | Cyst wall protein 2 [Giardia lamblia] | 710 | 362 | 34.0 | 26.3 |
| p-cluster593714 | 28065 | Dynamin-like protein [Giardia intestinalis] | 1537 | 732 | 31.5 | 20.4 |
| p-cluster094690 | 28068 | GLP [Giardia lamblia] | 2087 | 445 | 39.6 | 2.5 |
| p-cluster479841 | 28073 | Cruzipain [Trypanosoma cruzi] | 1836 | 383 | 35.5 | 11.1 |
| p-cluster198255 | 28596 | Mitogen-activated protein kinase 13 | 204 | 365 | 44.3 | 38.7 |
| p-cluster240502 | 28596 | Mitogen-activated protein kinase 13 | 540 | 365 | 30.1 | 34.6 |
| p-cluster291907 | 28596 | Mitogen-activated protein kinase 13 | 544 | 365 | 34.2 | 20.2 |
| p-cluster069608 | 30732 | Calglandulin | 164 | 156 | 33.1 | 89.6 |
| p-cluster508880 | 30773 | Venom serine protease or Allergen | 1403 | 277 | 30.3 | 11.6 |
| p-cluster496872 | 31481 | Venom prothrombin activator | 1149 | 467 | 28.8 | 19.0 |
| p-cluster707160 | 31481 | Venom prothrombin activator | 591 | 467 | 32.4 | 32.3 |
